# Development, Validation and Deployment of a Real Time 30 Day Hospital Readmission Risk Assessment Tool in the Maine Healthcare Information Exchange

**DOI:** 10.1371/journal.pone.0140271

**Published:** 2015-10-08

**Authors:** Shiying Hao, Yue Wang, Bo Jin, Andrew Young Shin, Chunqing Zhu, Min Huang, Le Zheng, Jin Luo, Zhongkai Hu, Changlin Fu, Dorothy Dai, Yicheng Wang, Devore S. Culver, Shaun T. Alfreds, Todd Rogow, Frank Stearns, Karl G. Sylvester, Eric Widen, Xuefeng B. Ling

**Affiliations:** 1 HBI Solutions Inc., Palo Alto, California, United States of America; 2 Departments of Surgery, Stanford University, Stanford, California, United States of America; 3 Departments of Pediatrics, Stanford University, Stanford, California, United States of America; 4 Shanghai Children's Hospital, Shanghai Jiao Tong University, Shanghai, China; 5 HealthInfoNet, Portland, Maine, United States of America; D'or Institute of Research and Education, BRAZIL

## Abstract

**Objectives:**

Identifying patients at risk of a 30-day readmission can help providers design interventions, and provide targeted care to improve clinical effectiveness. This study developed a risk model to predict a 30-day inpatient hospital readmission for patients in Maine, across all payers, all diseases and all demographic groups.

**Methods:**

Our objective was to develop a model to determine the risk for inpatient hospital readmission within 30 days post discharge. All patients within the Maine Health Information Exchange (HIE) system were included. The model was retrospectively developed on inpatient encounters between January 1, 2012 to December 31, 2012 from 24 randomly chosen hospitals, and then prospectively validated on inpatient encounters from January 1, 2013 to December 31, 2013 using all HIE patients.

**Results:**

A risk assessment tool partitioned the entire HIE population into subgroups that corresponded to probability of hospital readmission as determined by a corresponding positive predictive value (PPV). An overall model c-statistic of 0.72 was achieved. The total 30-day readmission rates in low (score of 0–30), intermediate (score of 30–70) and high (score of 70–100) risk groupings were 8.67%, 24.10% and 74.10%, respectively. A time to event analysis revealed the higher risk groups readmitted to a hospital earlier than the lower risk groups. Six high-risk patient subgroup patterns were revealed through unsupervised clustering. Our model was successfully integrated into the statewide HIE to identify patient readmission risk upon admission and daily during hospitalization or for 30 days subsequently, providing daily risk score updates.

**Conclusions:**

The risk model was validated as an effective tool for predicting 30-day readmissions for patients across all payer, disease and demographic groups within the Maine HIE. Exposing the key clinical, demographic and utilization profiles driving each patient’s risk of readmission score may be useful to providers in developing individualized post discharge care plans.

## Introduction

From 2007 to 2010, the national inpatient 30 day post discharge readmission rate remained relatively unchanged and included approximately 18 percent of Medicare patients. Medicare hospital readmissions cost the US taxpayer 15 billion dollars annually [1, 2]. Causes of potentially preventable hospital readmissions have been consistently identified to include premature discharge from the hospital, lack of resources for post discharge treatment, and insufficient provider consultation [3]. Accordingly, unplanned hospital readmissions impose a heavy burden to the US health care system, and serve as an overall indicator of poor quality [4, 5]. As a result, the Centers for Medicare and Medicaid Services (CMS) established a Hospital Readmission Reduction Program that defines a readmission as an admission to the hospital within 30 days post discharge from any hospital [6, 7]. Under reimbursement programs established by CMS in 2012, hospitals with high readmission rates for selected chronic diseases are penalized a percentage of overall reimbursement [8]. In an effort to prevent unwanted and avoidable hospital readmissions, it is first necessary to develop tools for actionable risk assessment and prediction, such that accountable healthcare stakeholders can target resources to those populations likely to yield the most benefit.

Previous studies addressing risk of readmission proposed risk models for specific disease cohorts including heart failure [9–13], acute myocardial infarction [13, 14], and pneumonia [13, 15], or for specific patient demographics including the elderly [16], children [17] or veterans [18]. The limitations in these models are apparent when considered across a population that includes all payers, all diseases and all demographics. Many prior studies lacked prospective testing and validation, reporting their performance on retrospective cohorts only [19]. Consequently, current models are of limited use for population health and case management tasked with reducing the readmission rate among the most vulnerable. The variability in research methods and results regarding the development of 30-day readmission risk models supports the need for ongoing development of more robust methods [20].

The increasing adoption of electronic medical record (EMR) systems and the development of health information exchanges (HIEs) have together facilitated the availability of detailed longitudinal patient medical histories to support the development of new methods to address patient population risk assessment. We have previously applied machine learning approaches to a statewide HIE database to predict emergency department 30 day revisits [21]. Our hypothesis is that population risk assessment can be rendered more accurate and actionable through the novel application of advanced machine learning with detailed and longitudinal clinical records. The specific objective in this study was to develop a model for predicting all-cause inpatient readmission risk in the HIE system within 30 days post discharge.

## Methods

### Ethics statements

This work was done under a business associate agreement between HealthInfoNet (HIN), which operates the Maine Health Information Exchange, and HBI Solutions, Inc. Data use was governed by the Business Associate Agreement (BAA) between HIN and HBI. No Protected Health Information (PHI) was released for the purpose of this research. HBI implemented their risk models within the Maine HIE, and the Maine HIE provides its members access to the risk scores through its secure platform. Since this study analyzed de-identified patient data, the Stanford University Institutional Review Board considered it exempt (October 16, 2014).

### Population

We set to develop a 30-day inpatient readmission risk model utilizing all inpatient encounters from the HIE member hospitals. The qualification standard was that the patient should be alive at the time of discharge, and not transferred to another acute care facility within the time frame of the cohort. The number of inpatient encounters in the total population was 211,232 from January 1, 2012 to December 31, 2013.

### Data acquisition

An enterprise data warehouse, consisting of the Maine HIE aggregated patient histories, was developed as previously described [21, 22] (See S1 File). A sequential staging data warehouse was utilized to extract, transform and load all EMR data from the HIE system. Data cleaning and integration was applied for handling errors and data quality issues (See S2 File) [23]. Subsequently, an analysis database of all data attributes was built based on the staging database for the machine learning process.

### Study design

**Fig 1:**
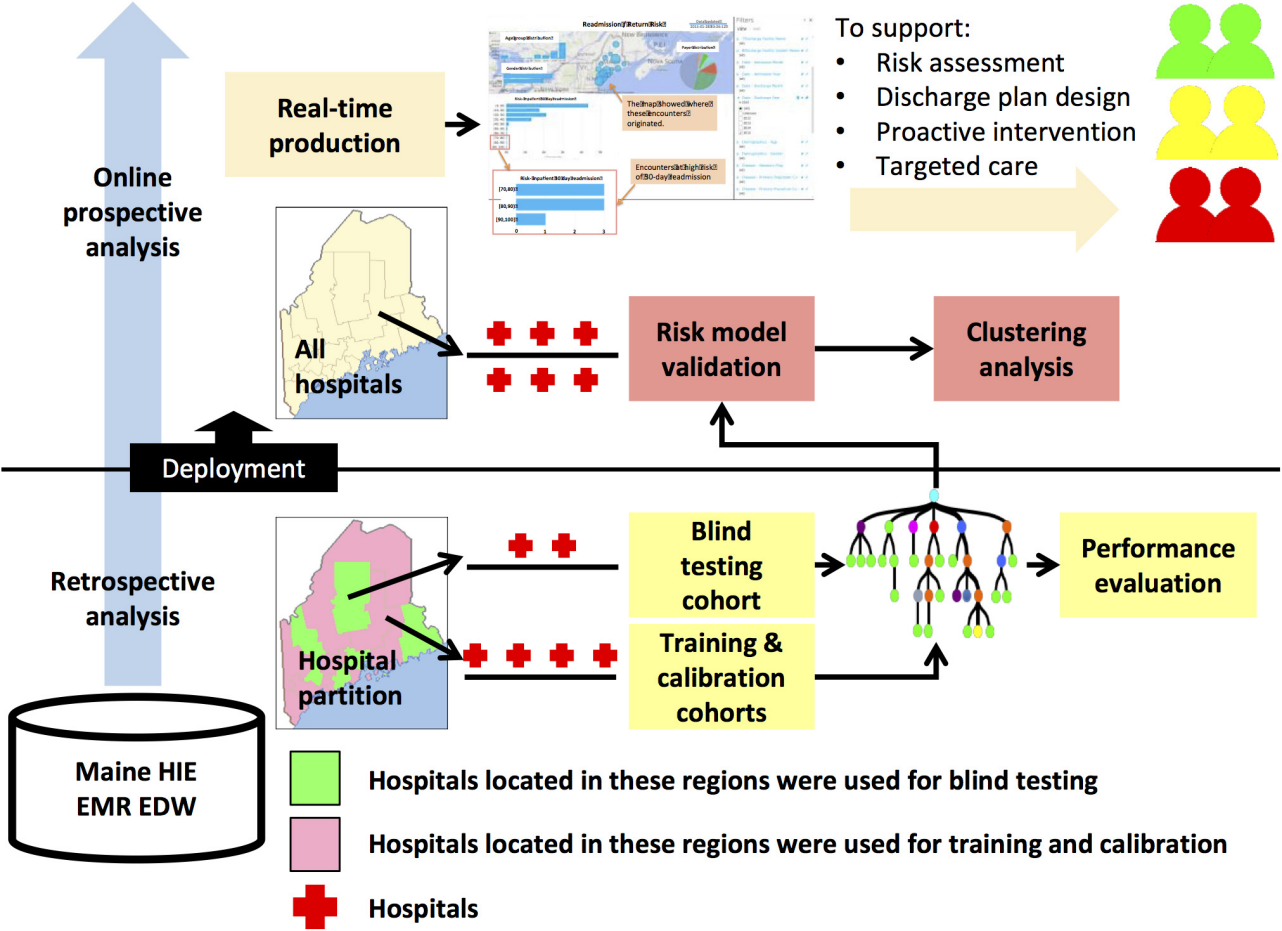
Study design for modeling the risk of an inpatient hospital readmission 30 days post discharge. There were three steps in model development: 1) two independent cohorts were constructed for retrospective modeling and prospective validation; 2) the retrospective cohort was split into two subgroups with each incorporating non-overlapped care facilities. The first subgroup was further split into model training and calibration sub cohorts, and the second subgroup was used as the blind-test cohort; and 3) the model was validated using the prospective cohort. Unsupervised clustering pattern analysis that included demographic and clinical data was performed. The prospectively validated model was then deployed in production to support healthcare quality monitoring and improvement efforts.

The 30-day inpatient readmission algorithm was built and validated in two phases (Fig 1): 1) retrospective modeling, in which the model was trained, calibrated and tested in three separate sub-cohorts to develop a risk scoring readmission metric; 2) prospective analysis, in which the model was validated to gauge its prospective performance, and to reveal high-risk sub-population clustering patterns. R statistical computing software was used for model development and validation.

### Cohort construction

There were a total of 74,484 inpatient encounters from January 1, 2012 to December 31, 2012 from 24 independent hospitals employed in the retrospective cohort (S1 Fig, top). In the prospective analysis phase, a total of 118,951 encounters between January 1, 2013 and December 31, 2013 involving all HIE hospitals were included (S1 Fig, bottom). The prospective cohort represented independent encounters from the retrospective cohort. Retrospective and prospective patients shared similar demographics (S1 Table). For patients who had an inpatient encounter, all of the patients’ past one-year clinical histories before the discharge date were utilized in the subsequent statistical learning.

For exploratory data analysis (S2 Fig), we profiled the retrospective readmissions to establish the prevalence of past one-year inpatient admissions and the presence of chronic disease diagnoses. This analysis showed that the inpatient history and the counts of chronic diseases were strongly associated with the risk of future inpatient readmissions, providing a grouping method to develop four specific models. The four models were developed, calibrated, and validated in parallel in the modeling process based on the four sub-cohorts demonstrated in S1 Fig, which were groups with chronic diseases and inpatient history, with chronic diseases but no inpatient history, with inpatient history but no chronic disease, and with no chronic disease nor inpatient history, respectively.

### Pre-processing

Initially, a total of 14,680 features were extracted from the enterprise data warehouse. Considering that some features were redundant or uninformative to the statistical learning, we applied variance minimization criterion [24] to determine the discriminant features. As a result, 2,000 features were selected for risk modeling.

### Retrospective cohort subgroups

In the retrospective analysis, HIE patient inpatient encounters were partitioned into two subgroups according to the associated hospital encounters, balancing the monthly inpatient readmission volume and rate between the two subgroups. The first subgroup of patients was further split into training and calibration sub cohorts. The second subgroup was used as a blind-test sub cohort. Together, the three sub cohorts were utilized for retrospective model development.

### Retrospective modeling steps

1) Using the 2,000 qualified features, the number of days post discharge as the time variable, and readmission as the outcome event, we conducted a survival analysis by random forest [25] that represented a forest ensemble learning method trained in a bootstrapping manner.

First, a general technique of bootstrap aggregating (bagging) was applied for building 300 decision trees by repeatedly and randomly resampling the training cohort with replacement, and voting the trees for a consensus prediction. Second, the survival trees were grown based on the randomly selected predictors via Log-rank survival splitting rule on each survival tree node that maximizes survival differences across daughter nodes.

$$\text{F}\left(\text{x},\;\text{c}\right)=\frac{\sum_{\text{i}=1}^{\text{N}}\left({\text{d}}_{\text{i},1}-{\text{Y}}_{\text{i},1}\frac{{\text{d}}_{\text{i}}}{{\text{Y}}_{\text{i}}}\right)}{\sqrt{\sum_{\text{i}=1}^{\text{N}}\frac{{\text{Y}}_{\text{i},1}}{{\text{Y}}_{\text{i}}}\left(1-\frac{{\text{Y}}_{\text{i},1}}{{\text{Y}}_{\text{i}}}\right)\left(\frac{{\text{Y}}_{\text{i}}-{\text{d}}_{\text{i}}}{{\text{Y}}_{\text{i}}-1}\right){\text{d}}_{\text{i}}}}$$ (1)

Here, c is the split value for predictor x; d_i,j_ and Y_i,j_ for the node h equal the number of patients that had a readmission event in t_i_ day after discharge and who never come back in t_i_ day after discharge for the daughter nodes j = 1,2. Hence, Y_i,1_ = #{T_l_ ≥ t_i_ & x_l_ ≤ c} and Y_i,2_ = #{T_l_ ≥ t_i_ & x_l_ > c}, where T_l_ is the days for an individual patient l return to a hospital after discharge. The value |F(x, c)| is the measure of node separation, the greater difference between case and control groups and the better the split for the predictor was realized. Therefore, the optimized predictor x^*^ and split value c^*^ at the node h is determined by maximizing the |F(x^*^, c^*^)| such that |F(x^*^, c^*^)| ≥ |F(x, c)| for all x and c. For each tree the maximum terminal node size is 1.

Third, an ensemble cumulative hazard estimate by combining information from the survival trees so that each individual will be assigned one estimate.

$$\hat{\text{H}}\left(\text{t}|{\text{x}}_{\text{i}}\right)=-\text{log}\;\text{S}\left(\text{t}|{\text{x}}_{\text{i}}\right)$$ (2)

Where $\hat{\text{H}}\left(\text{t}|{\text{x}}_{\text{i}}\right)$ is the cumulative hazard estimate computed for terminal node for each predictor x_i_ for individual sample i drop down into in the tree; S(t|x_i_) is the survival function expressed as: S(t|x_i_) = P(T > t|x_i_), with T representing a readmission event happened in T days post discharge.

To derive an individual estimate for all trees, an ensemble average for all tree cumulative hazard estimate score was computed.

$${\hat{\text{H}}}_{\text{e}}\left(\text{t}|{\text{x}}_{\text{i}}\right)=\frac{1}{\text{ntree}}\sum_{\text{b}=1}^{\text{ntree}}\;{\hat{\text{H}}}_{\text{b}}\left(\text{t}|{\text{x}}_{\text{i}}\right)$$ (3)

Here, b denotes the individual tree and ntree is the number of trees in the survival forest.

2) A calibrating cohort was used to calibrate the predictive scoring threshold ${\hat{\text{H}}}_{\text{e}}\left(\text{t}|{\text{x}}_{\text{i}}\right)$ to create a risk measure of 0–100 for each individual encounter. Applying the model developed with each of the four training sub-cohorts to each encounter in the corresponding calibrating sub-cohort, the derived cumulative hazard score ${\hat{\text{H}}}_{\text{e}}\left(\text{t}|{\text{x}}_{\text{i}}\right)$ was ranked. Each of the four sub-cohorts had different scales of ${\hat{\text{H}}}_{\text{e}}\left(\text{t}|{\text{x}}_{\text{i}}\right)$ derived using a separate model, making them not comparable to each other. In order to make the model outcomes from different sub-cohorts comparable to each other, a 0–100 risk measure was derived from the positive predictive values (PPVs) associating with each ${\hat{\text{H}}}_{\text{e}}\left(\text{t}|{\text{x}}_{\text{i}}\right)$, which provided a universal, standardized measure of readmission risk for all the samples from all sub-cohorts. PPV for each estimate was calculated as the proportion of samples having readmission in a subset of sample having ${\hat{\text{H}}}_{\text{e}}\left(\text{t}|{\text{x}}_{\text{i}}\right)$ higher than that estimate. The mapping function between the risk measure and the cumulative hazard estimate ${\hat{\text{H}}}_{\text{e}}\left(\text{t}|{\text{x}}_{\text{i}}\right)$ for each of four sub-cohorts was shown on S3 Fig. The risk measure thus described the probability that a patient will have an inpatient readmission within 30 days post discharge.

Based on the mapping, we defined three risk groups: High (score ≥ 70), Low (score < 30), and Intermediate (30 ≤ score < 70). Our analysis therefore produced two risk measures: a continuous risk score ranging from 0 to 100, and a categorical risk defined by three levels. The former was applied for numerical performance tests while the latter was used for stratified analysis. The thresholds (30 and 70) were chosen arbitrarily.

3) During the first round of the survival analysis, the number of features utilized was 2,000 as selected by variance minimization criterion. In the second round, we initiated modeling by utilizing the top 10 features of computed importance, and then iteratively built models by adding additional features of significance. During iterative modeling, optimum performance was determined for each step according to the sensitivity, specificity and PPV. As a result, 243 features were identified as the best performing features for risk assessment for each patient (Table 1) through a feature combination process utilizing the four sub-cohort models (S1 Fig, top). Top 10 important features in each sub-cohort models were displayed on S4 Fig.

**Table 1:** The final list of features in the model after 2 rounds of feature selections.

Feature group	Number	Feature description (past 12 month clinical histories)
**Encounter history**	118	Visit counts of different encounter types (E/O/I/P/R) ^a^, the accumulated length of hospitalized stay, counts of historical chronic disease diagnoses, counts of total and non redundant total radiographic, counts of total and non redundant laboratory tests, and counts of total and non redundant outpatient prescriptions
**Demographics**	4	Gender, income, education, payer, and age group that is defined by age at IP admission (0, 1–5yr, 6–12yr, 13–18yr, 19–34yr, 35–49yr, 50–65yr, 65+yr) ^b^
**Radiology**	2	Different radiology tests
**Payer**	1	Different payer information
**Chronic disease condition**	19	Counts for chronic diseases
**Diagnosis**	4	Counts for primary diagnosis and secondary diagnosis
**Laboratory test**	24	Counts for different laboratory test results
**Outpatient prescriptions**	71	Counts for different outpatient prescriptions

^a^Encounter type descriptions: E-Emergency, O-Outpatient, I-Inpatient, P-Pre admission, R-Recurring admission

^b^yr-year

### Prospective analysis

The model developed in the retrospective phase was prospectively validated during the 2013 calendar year from the HIE data warehouse. The risk-stratified 30-day readmission statistics as well as the time-to-event curve were used to gauge model performance that was then compared with prior similar model studies [19, 26–28]. In order to derive a better understanding of the high-risk population’s characteristics, we determined patterns of clinical and demographic features by applying an unsupervised learning approach. First, we applied a principal component analysis (PCA) [29] to the selected features of all high-risk encounters identified by our risk prediction model to derive their factor scores, of which the first 2 dimensions were selected to represent each sample. Second, the K-means analysis discovered six clusters in which the patients shared similar 2-dimensional PCA scores.

The number of clusters (K = 6) was determined by observing change of the sum of squares of clusters as the number increased. In K-means analysis, the total within-cluster sum of squares (TWSS) was defined as the sum of the sum of squares in every cluster. When K = 1, all samples belonged to the same cluster, thus the TWSS equaled to the variance of the samples. The TWSS monotonically decreased to 0 as K increased to reach its maximum value, i.e. the number of samples, which was over-fitting. We measured the contribution of adding a new cluster as the reduction rate of TWSS:

$$R_{k}=\frac{TWSS_{k-1}-TWSS_{k}}{TWSS_{k-1}}$$ (4)

We determined K to achieve the balance between the over-fitting and variance modeling:

$$K={\text{max}}_{k}\left\{k,\;R_{k}>0.2\right\}$$ (5)

The TWSS and R_k_ with respect to K is shown in S5 Fig. R_k_ reached its peak at K = 6, so K = 6 was selected for our subsequent clustering analysis. The within-cluster sum of squares in clusters #1 to #6 (See S6 Fig) were: 47.2, 68.7, 102.7, 85.2, 53.8, and 54.8.

Visualization of the clustering results demonstrated that the 6 clusters represented unique patterns of the corresponding sub-populations within the high-risk patient population.

## Results

**Fig 2:**
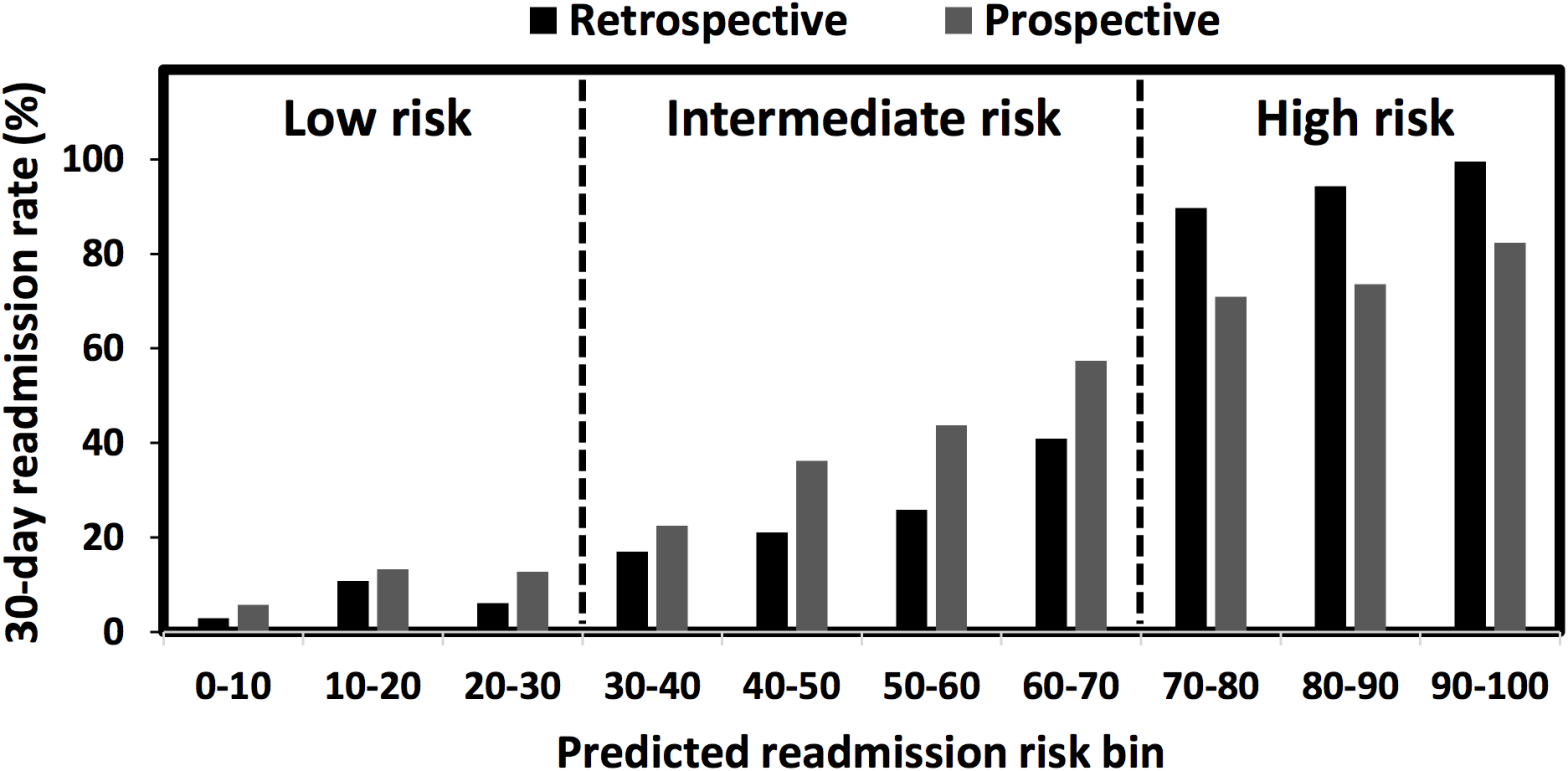
Retrospective and prospective results of the 30-day readmission risk stratification. 30-day readmission rates were measured in 10 risk bins by intervals of 10. The risk metric was divided into three regions: low (0–30), intermediate (30–70), and high (70–100).

The model was evaluated on an independent cohort by using the rate of readmissions stratified by the risk level and PPV. The continuous scores measuring the 30-day readmission risk were converted into 10 risk bins ranging from 0–100, with the 30-day readmission rate (i.e. PPV) summarized for each bin (Fig 2). From the low risk to high-risk groups, both the retrospective and prospective 30-day readmission rates increased almost monotonically, revealing that the risk stratification model provided a reasonable measure of 30-day readmission probability. The average 30-day readmission rates in low (score of 0–30), intermediate (score of 30–70) and high (score of 70–100) risk partitions were 8.67%, 24.10% and 74.10%, respectively. A 20% less readmission rate was found in the high-risk cohort comparing prospective to retrospective modeling periods, however the model maintained an impressive call rate identifying 74.10% of hospital readmissions in the high-risk group during prospective testing. The rate of readmission increased significantly with rising computed level of risk indicating the effectiveness of the model and case finding methodology.

**Fig 3:**
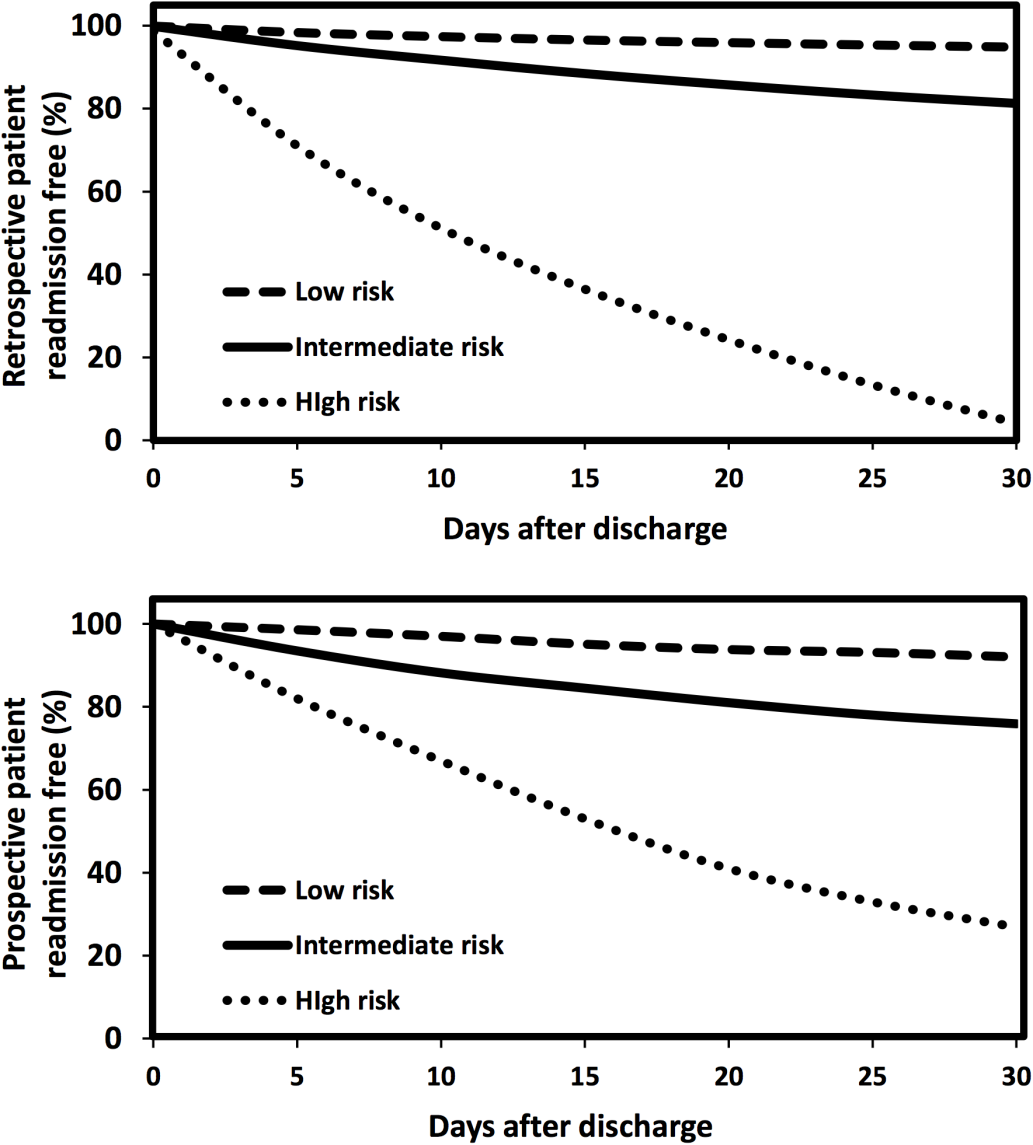
Time to event analysis on retrospective (top) and prospective cohorts. ‘Time to event’ graphic representation of the low-, intermediate-, and high-risk patients’ time to the next impending inpatient visit.

The time-to-event curves measuring the patient readmission free rate within 30 days after discharge (Fig 3) demonstrated that the readmission rate for high-risk patients was significantly higher at the same time point, compared with the rates for intermediate or low risk patients. The figure also showed that more than 50% of readmitted patients experienced readmission within 15 days post discharge in each risk level.

In order to explore the high-risk population for common clinical patterns, unsupervised learning of principal component analysis was applied to the prospective cohort. Six distinct patient subgroups were identified with the following characteristics (S6 Fig): 1) the largest cluster (#1, n = 1,036 encounters) was mainly occupied by younger patients (age range 0–35 years, 78.2%); 2) the smallest cluster (#6, n = 251 encounters) predominantly included senior patients (age range 65+ years, 96.1%); 3) most of the high-risk patients had one or more chronic disease diagnoses (89.8% and 62.3% in clusters #2 and #3, and 100% in clusters #4, #5 and #6, except in cluster #1 (59.9% with no chronic disease). The clinical pattern identification of these high-risk patients may allow providers and care managers to apply more targeted interventions to reduce the risk of readmission.

We compared our model to previous studies on 30-day readmission risk prediction (Table 2). The patient demographics, sample size, and model performance are illustrated in the table. Unlike many previous studies that focused on specific age, disease or payer groups from one or several care facilities, our study targeted a statewide population that included all HIE member hospitals, and all HIE patients with an acute care admission. The c-statistics of our predictive model were 0.86 for the retrospective cohort and 0.72 with the prospective cohort (S7 Fig), performing as well or better than similar studies that focused on specific patient groups. These findings demonstrate that comprehensive clinical data analysis can yield whole population models for risk assessment that are uniform and do not require *a priori* patient cohorting by chronic disease or other qualifiers.

**Table 2:** Comparison of our model with previous studies.

Study	Population demographics	Sample size (derivation and validation)	c-statistics
**Donzé, 2013 [28]**	Adult patient, 2009–2010	6,141 and 3,071	0.71
**Allison, 2014 [19]**	Patients discharged receiving outpatient parenteral antibiotic therapy, 2009–2011	782 and NA	0.61
**Eapen, 2013 [27]**	Heart failure (HF) patients ≥65 years of age, 2005–2009	70% and 30% of 30,828	0.59
**Vigod, 2015 [26]**	Adults discharged from an acute psychiatric unit, 2008–2011	32,749 and 32,750	0.63
**HIE model**	all age, all payer, all disease, 2012–2013	74,484 and 118,951	0.72

**Fig 4:**
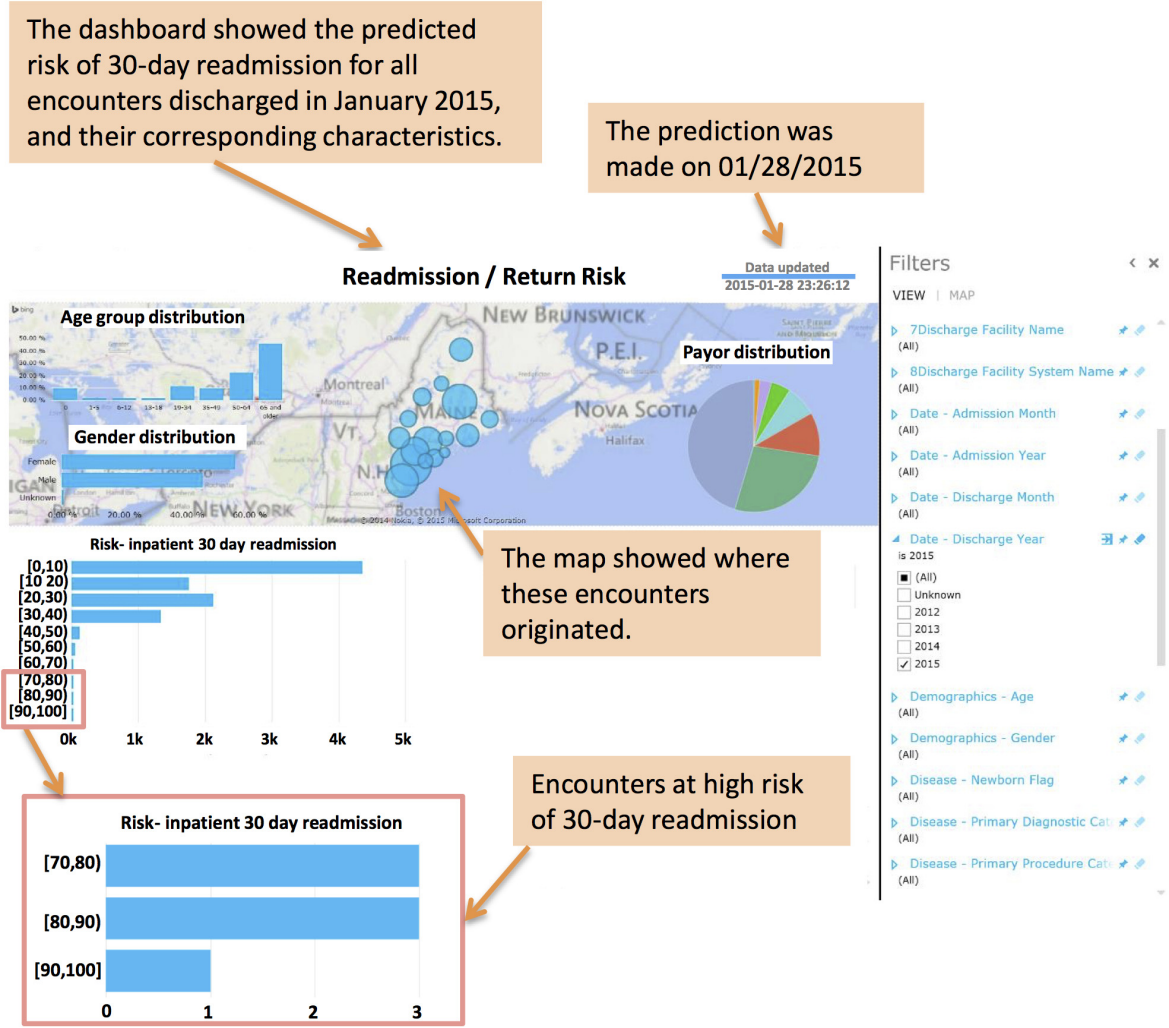
The deployment of the 30-day readmission risk model. The validated risk model was deployed via a real time provider portal that was integrated into the Maine HIE. The model and results are subject to continuous adaptation in response to EMR output on a daily basis. A screenshot: the real-time dashboard allowing for high-risk inpatient encounter identification and in support of targeted interventions is shown.

By integrating our algorithm into a HIE-supported online platform, patient risk scores can be updated on a daily basis for the population in Maine. Fig 4 shows the platform visualization where 30-day readmission risk screening was displayed for Jan 28, 2015, for all inpatients discharged within the previous 30 days. Additionally, statistics for the demographic and payer mix of the population were summarized on the dashboard.

## Discussion

Leveraging the vast number of EMR clinical features and encounters in the Maine HIE data warehouse, we developed and tested a clinical algorithm to predict the risk of readmission within 30 days post discharge for inpatients across the entire state population. Through the profiling of the comprehensive longitudinal clinical histories, the developed model and the derived risk scores facilitated active high-risk case finding and risk stratification of the patient population in Maine. The risk predictive analytics (prospective c-statistic: 0.72) for the entire population outperformed the results of prior specific patient cohort based studies [19, 26–28, 30–36]. The results of the prospective validation analysis demonstrate the robust reproducibility of our methods for the derivation of reliable risk assessment. Taken together, these results support the hypothesis that a 30-day readmission event, regardless of patient demographics and clinical conditions, can be accurately determined using the clinical data managed in a statewide HIE database.

A couple of limitations of this study were noticed. There was a drop in model accuracy from the retrospective to prospective analysis (Figs 2 and 3). It is to be expected, due to over fitting, that our model will perform less well on the prospective data set than on the retrospective data set used for fitting. To avoid over fitting, we sub-divided the retrospective training set into training and calibrating sets, which can indicate when further training is not resulting in better generalization. It is possible that our prospective cohort, even with the similar demographics profiles as the retrospective one (S1 Table), had evolving clinical profiles, leading to differentiated feature networks driving the readmission to cause prospective performance degradation in risk prediction. Therefore, predictive analytics errors, when applied as a decision-assisting tool in clinical sites, would result in inappropriate post-discharge plans. To overcome over fitting and longitudinal data evolving issues, the data quality and integrity would be closed monitored and the model would be re-trained periodically with new data contents and newly-added attributes. In addition, care providers’ feedback will be collected to identify the performance variations as a function of the longitudinal time frame and geographic locations. These multi-faceted efforts should enhance our knowledge base and help to identify more genuine clinical drivers of readmission that would not be explicitly revealed in the EMR data mining process. Another limitation was that while HIE data represents an ideal source of community-wide/regional patient data, operational HIEs are not present in all States. Although the samples collected from the HIE for our study were with all ages, all payers and all diseases in Maine State, they may have an unexpected bias and may not exactly match the nationwide population characteristics and hospital visit trends. After overcoming these limitations, our predictive model will be improved with a broader applicability in health care globally.

The 30-day readmission rate is a useful indicator of increased risks of adverse outcomes [37]. Evidence suggests that well-organized post-discharge interventions targeting high-risk patients can result in a decreased rate of readmissions and therefore significant cost savings [38, 39]. However, most current risk assessment tools are not automated and require manual collection of data elements for analysis. Moreover, most current approaches to high-risk assessments are not done in real time. Through the integration of our case finding algorithms into a statewide HIE, scores were produced (Fig 4) on any patient upon admission and updated daily. Along with the risk scores, providers can develop, in real time, evidence based intervention plans that may benefit the patient in their post discharge care: these include interventions identified by the Project Boost effort. Project Boost (Better Outcomes for Older adults through Safe Transitions) effort is a mentored implementation program to improve the care of patients as they transition from hospital to home, by identifying patients with a high risk of readmission and offering targeted specific interventions to reduce the adverse outcomes and 30-day readmission rates. Studies showed that readmissions were decreased dramatically in hospitals where Project Boost tool was implemented [40, 41]. Based on Project Boost guidelines, patients with multiple complex medications (clusters #2–6) will benefit from interventions that eliminate unnecessary medications and improve medication compliance; older adults (clusters #3, 4, and 6) will benefit from home services and follow-up calls; and patients that have multiple chronic diseases (clusters #4–6) will benefit from education to understand their specific care goals and the signals to understand if these goals are met. These interventions, guided by our automated risk measures, can help clinicians spend more time applying targeted care to the appropriate patients and less time performing manual risk assessments. We believe that the real time, automated availability of risk assessment in tandem with personalized evidence based interventions will facilitate timely post-discharge planning that will lead to the avoidance of unwanted readmissions and increased costs. The utilization of this type of dynamic risk assessment will further facilitate the ongoing calibration of population risk assessment as post-discharge planning and case management interventions are formulated and tested.

The unsupervised learning of the high-risk patients’ profiles resulted in several data driven clusters with similar patterns. Not surprisingly, the incidence and types of chronic diseases within the high-risk population were two of the primary drivers of high-risk that were similar among sub-groups. Perhaps surprisingly, the largest overall sub-group was comprised of mostly younger adults without chronic disease. Observations such as these suggest that a one size fits all approach to case management targeting the avoidance of readmission is likely insufficient as each of these sub-groups have unique characteristics that suggest unique post-discharge needs. It is intriguing to speculate that this type of analysis could be used for a more personalized or precise approach to readmission prevention that would be amenable to ongoing adjustment and adaptation according to ongoing success and failures to prevent readmission. Since the risk assessment was successfully deployed within the HIE and is made available on a real time basis, the operational advantage of the presented tool will allow each planned discharge to be carefully evaluated to determine the necessity of continued hospitalization, balanced against the cost of a possible readmission. Accordingly, real time operational solutions such as those presented here are a necessary step in improving patient care.

## Conclusion

A risk assessment tool for predicting 30-day hospital readmissions was successfully developed, tested and deployed as a component of a real time HIE analytic platform. The advantages of the current tool include the prospective validation on a patient population that includes all payers, all diseases and all age groups. The identification of high-risk patients in real time can act as an early warning system that can drive timely care interventions, reduce readmissions, provide for safer transitions of care, and lower costs.

## Supporting Information

S1 Fig Cohort construction to support retrospective (Top) and prospective (Bottom) analyses. The retrospective cohort was further divided into train, calibration and blind test sub-cohorts.
(DOCX)

S2 Fig Exploratory data analysis correlating the inpatient readmission with inpatient history (Top) and number of chronic diseases (Bottom). The left and right y-axis represent the number of inpatient encounters and the rate of 30-day readmission, respectively. The x-axis indicates the total number of inpatient admissions (Top) and chronic diseases (Bottom) a patient had during the 12-month period before a discharge.
(DOCX)

S3 Fig Calibration plots showing the one-to-one mapping from cumulative hazard estimate to 0–100 risk measure, with four sub-cohorts, respectively. (DOCX)

S4 Fig Variable importance plots of four models developed with four sub-cohorts in parallel. Top 10 variables were displayed on each plot. Importance for each variable was measured by the increase of mean square error (MSE) of prediction by permuting that variable.
(DOCX)

S5 Fig The total within-cluster sum of squares (TWSS) and its changing rate (R_k_) as a function of the number of clusters in PCA analysis. (DOCX)

S6 Fig The prospective unsupervised learning of high-risk encounters. Summary of clinical patterns in each cluster is shown in (A)-(D). The y-axes stand for (A) the average number of lab tests, radiographic studies, and medications; (B) the average number of chronic diseases; (C) the percentage of three age groups, and (D) the percentage of chronic diseases.
(DOCX)

S7 Fig Binary classification performance of risk scores with retrospective cohort and prospective cohort, respectively. (DOCX)

S1 File Data warehouse. (DOCX)

S2 File Missing data handling. (DOCX)

S1 Table Patient characteristics. (DOCX)
