# Supplementary material for: Development, Validation and Deployment of a Real Time 30 Day Hospital Readmission Risk Assessment Tool in the Maine Healthcare Information Exchange
Source: PLoS One. 2015 Oct 8;10(10):e0140271. doi: 10.1371/journal.pone.0140271 (PMC4598005; doi:10.1371/journal.pone.0140271)
Supplement: S1 Fig — The retrospective cohort was further divided into train, calibration and blind test sub-cohorts. (DOCX) [file pone.0140271.s001.docx]

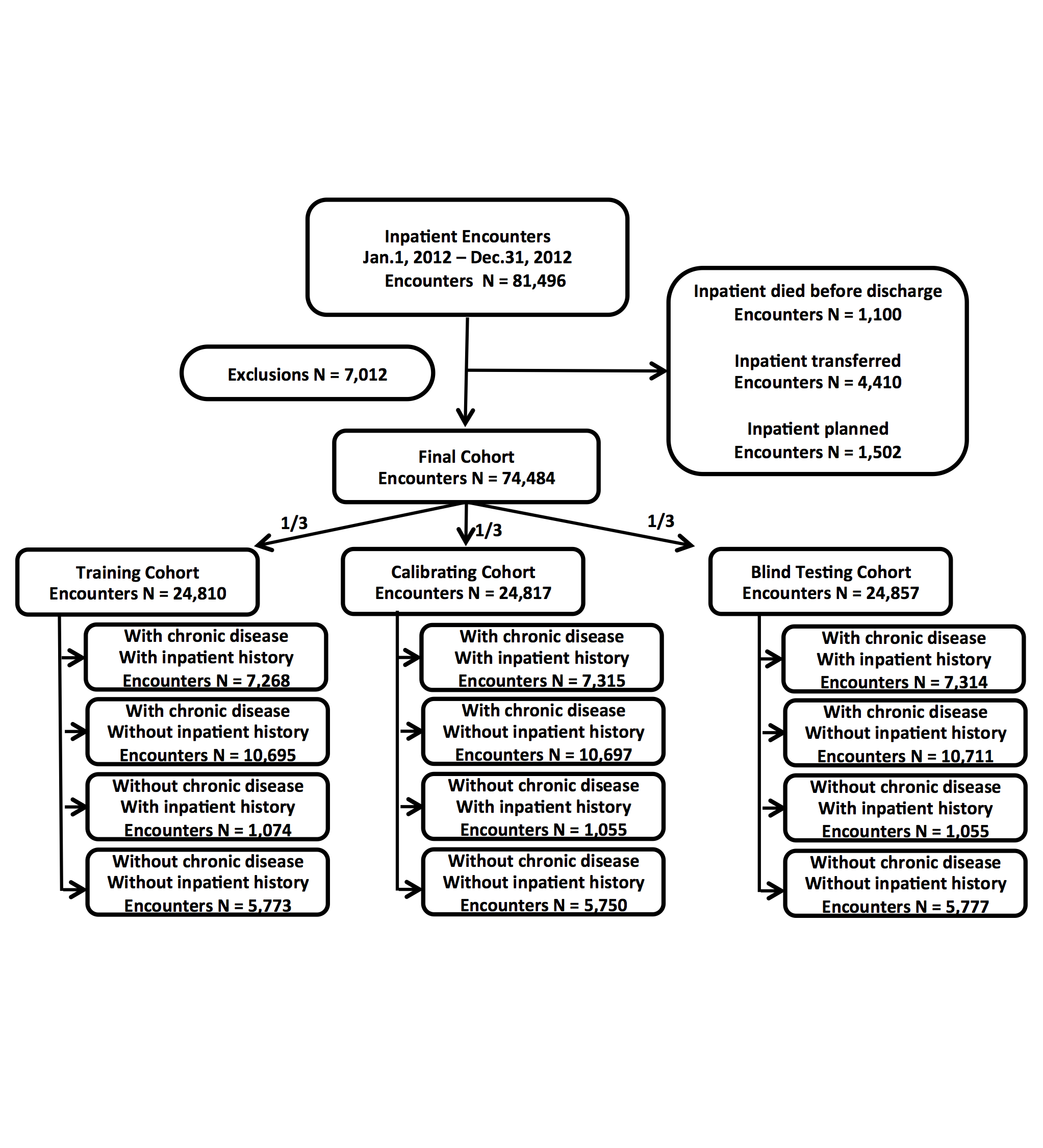


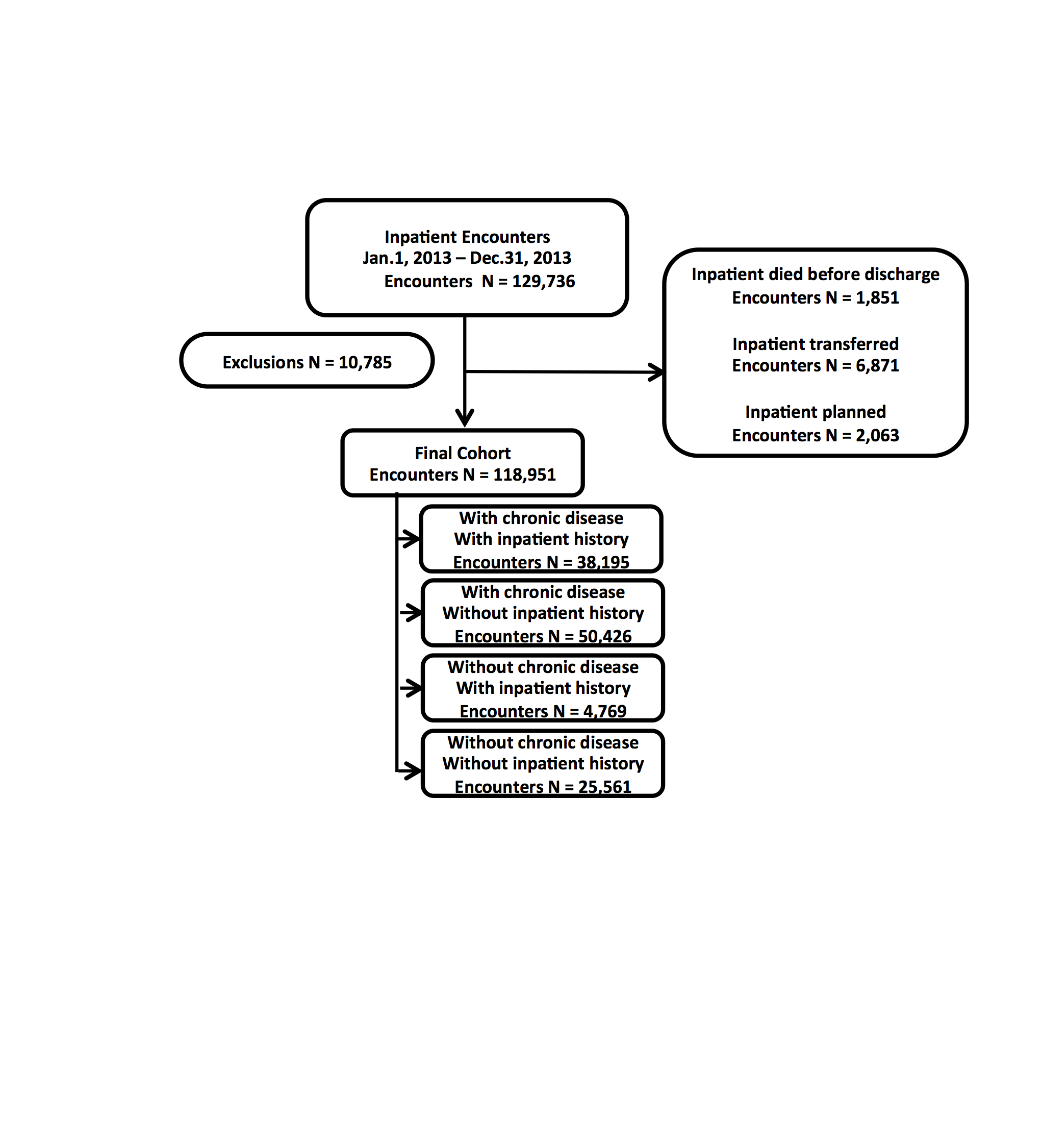


**S1 Fig. Cohort construction to support (Top) retrospective and (Bottom) prospective analyses.** The retrospective cohort was further divided into train, calibration and blind test sub-cohorts.
