# Supplementary material for: Development, Validation and Deployment of a Real Time 30 Day Hospital Readmission Risk Assessment Tool in the Maine Healthcare Information Exchange
Source: PLoS One. 2015 Oct 8;10(10):e0140271. doi: 10.1371/journal.pone.0140271 (PMC4598005; doi:10.1371/journal.pone.0140271)
Supplement: S2 Fig — The left and right y-axis represent the number of inpatient encounters and the rate of 30-day readmission, respectively. The x-axis indicates the total number of inpatient admissions (Top) and chronic diseases (Bottom) a patient had during the 12-month period before a discharge. (DOCX) [file pone.0140271.s002.docx]

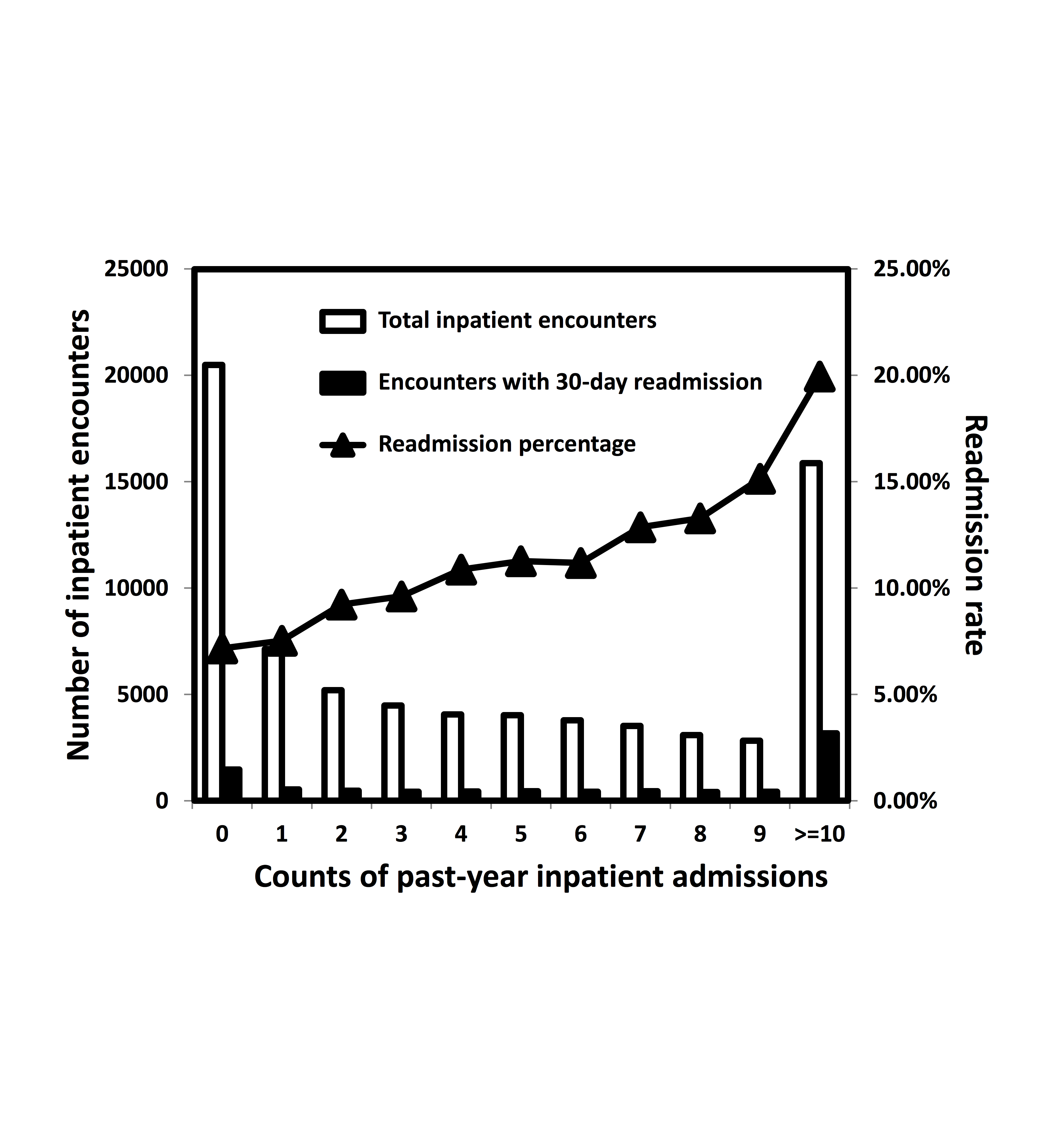


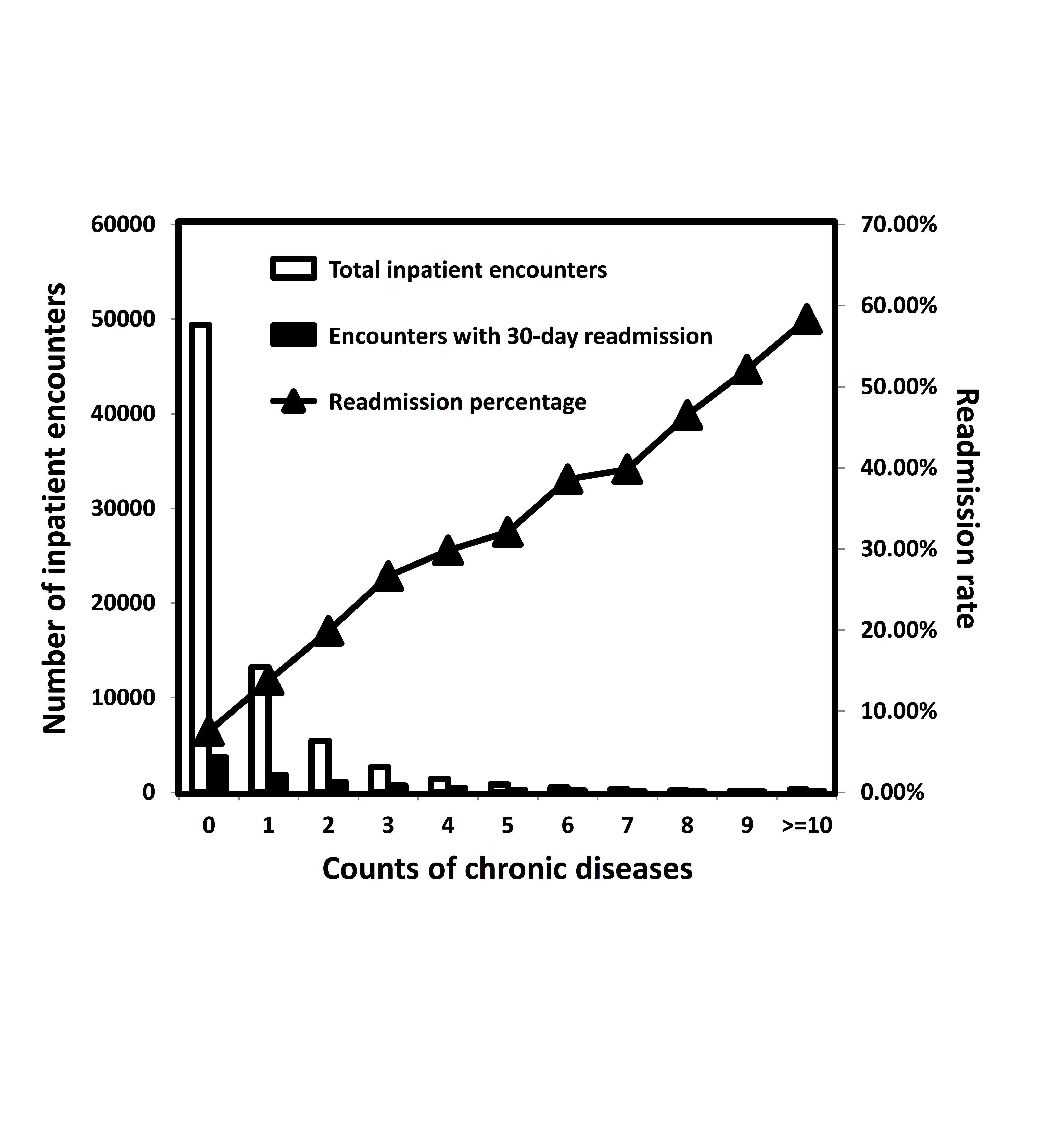


**S2 Fig.** **Exploratory data analysis correlating the inpatient readmission with inpatient history (Top) and number of chronic diseases (Bottom).** The left and right y-axis represent the number of inpatient encounters and the rate of 30-day readmission, respectively. The x-axis indicates the total number of inpatient admissions (Top) and chronic diseases (Bottom) a patient had during the 12-month period before a discharge.
