# Supplementary material for: Development, Validation and Deployment of a Real Time 30 Day Hospital Readmission Risk Assessment Tool in the Maine Healthcare Information Exchange
Source: PLoS One. 2015 Oct 8;10(10):e0140271. doi: 10.1371/journal.pone.0140271 (PMC4598005; doi:10.1371/journal.pone.0140271)
Supplement: S3 Fig — (DOCX) [file pone.0140271.s003.docx]

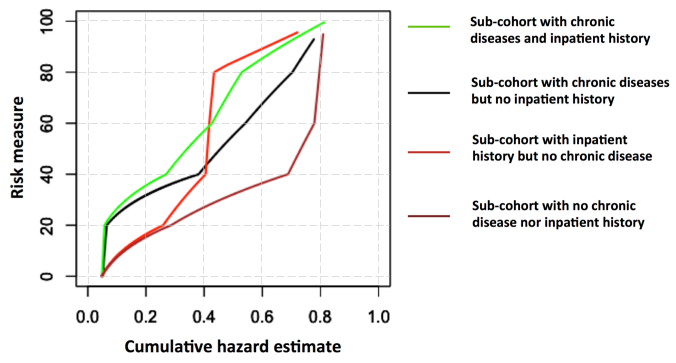


**S3 Fig. Calibration plots showing the one-to-one mapping from cumulative hazard estimate to 0-100 risk measure, with four sub-cohorts, respectively.**
