# Supplementary material for: Development, Validation and Deployment of a Real Time 30 Day Hospital Readmission Risk Assessment Tool in the Maine Healthcare Information Exchange
Source: PLoS One. 2015 Oct 8;10(10):e0140271. doi: 10.1371/journal.pone.0140271 (PMC4598005; doi:10.1371/journal.pone.0140271)
Supplement: S4 Fig — Top 10 variables were displayed on each plot. Importance for each variable was measured by the increase of mean square error (MSE) of prediction by permuting that variable. (DOCX) [file pone.0140271.s004.docx]

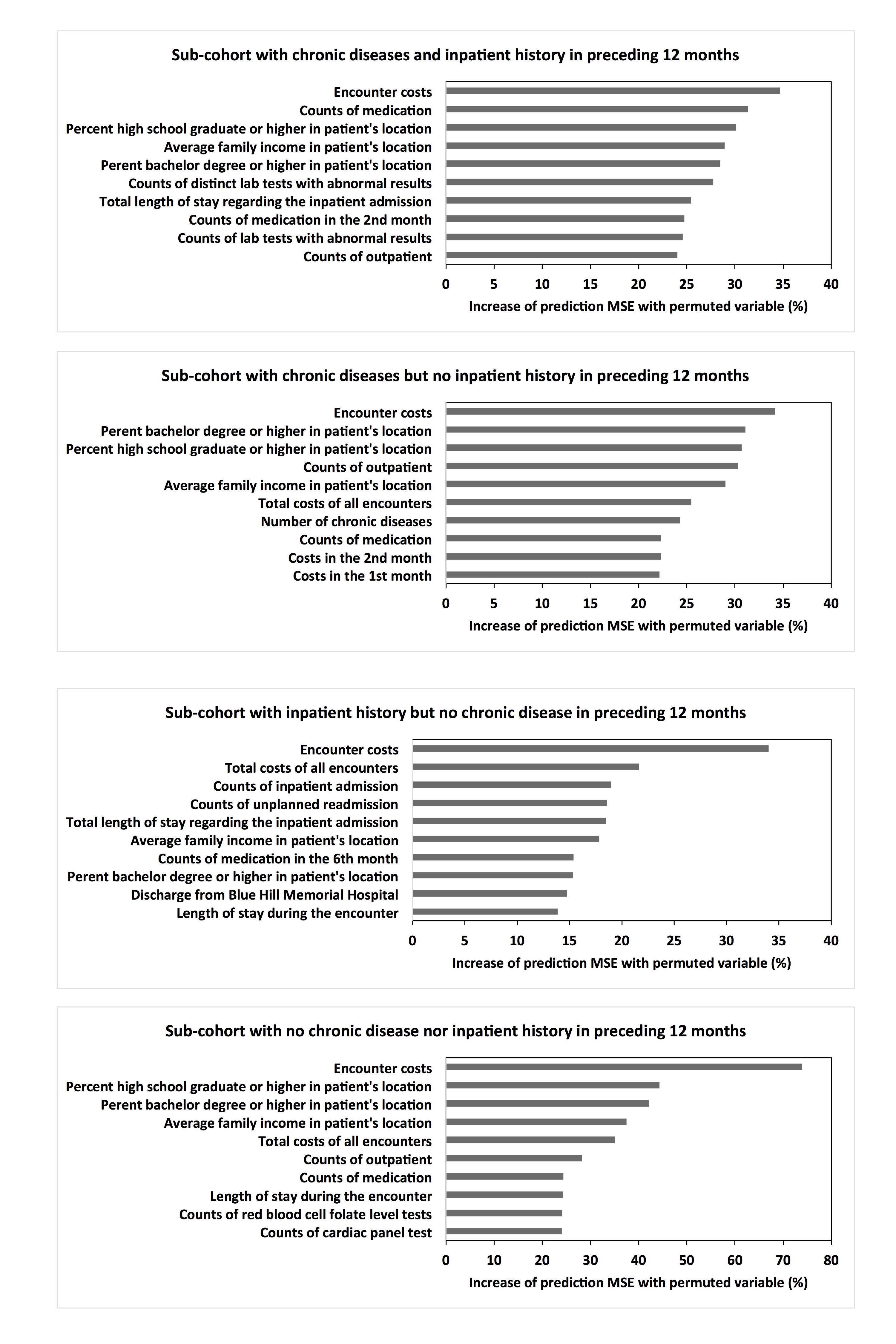


**S4 Fig.** **Variable importance plots of four models developed with four sub-cohorts in parallel.** Top 10 variables were displayed on each plot. Importance for each variable was measured by the increase of mean square error (MSE) of prediction by permuting that variable.
