# Supplementary material for: Development, Validation and Deployment of a Real Time 30 Day Hospital Readmission Risk Assessment Tool in the Maine Healthcare Information Exchange
Source: PLoS One. 2015 Oct 8;10(10):e0140271. doi: 10.1371/journal.pone.0140271 (PMC4598005; doi:10.1371/journal.pone.0140271)
Supplement: S5 Fig — (DOCX) [file pone.0140271.s005.docx]

**S5 Fig. The total within-cluster sum of squares (TWSS) and its changing rate (R_k_) as a function of the number of clusters in PCA analysis.**
