# Supplementary material for: Development, Validation and Deployment of a Real Time 30 Day Hospital Readmission Risk Assessment Tool in the Maine Healthcare Information Exchange
Source: PLoS One. 2015 Oct 8;10(10):e0140271. doi: 10.1371/journal.pone.0140271 (PMC4598005; doi:10.1371/journal.pone.0140271)
Supplement: S6 Fig — Summary of clinical patterns in each cluster is shown in (A)-(D). The y-axes stand for (A) the average number of lab tests, radiographic studies, and medications; (B) the average number of chronic diseases; (C) the percentage of three age groups, and (D) the percentage of chronic diseases. (DOCX) [file pone.0140271.s006.docx]

**
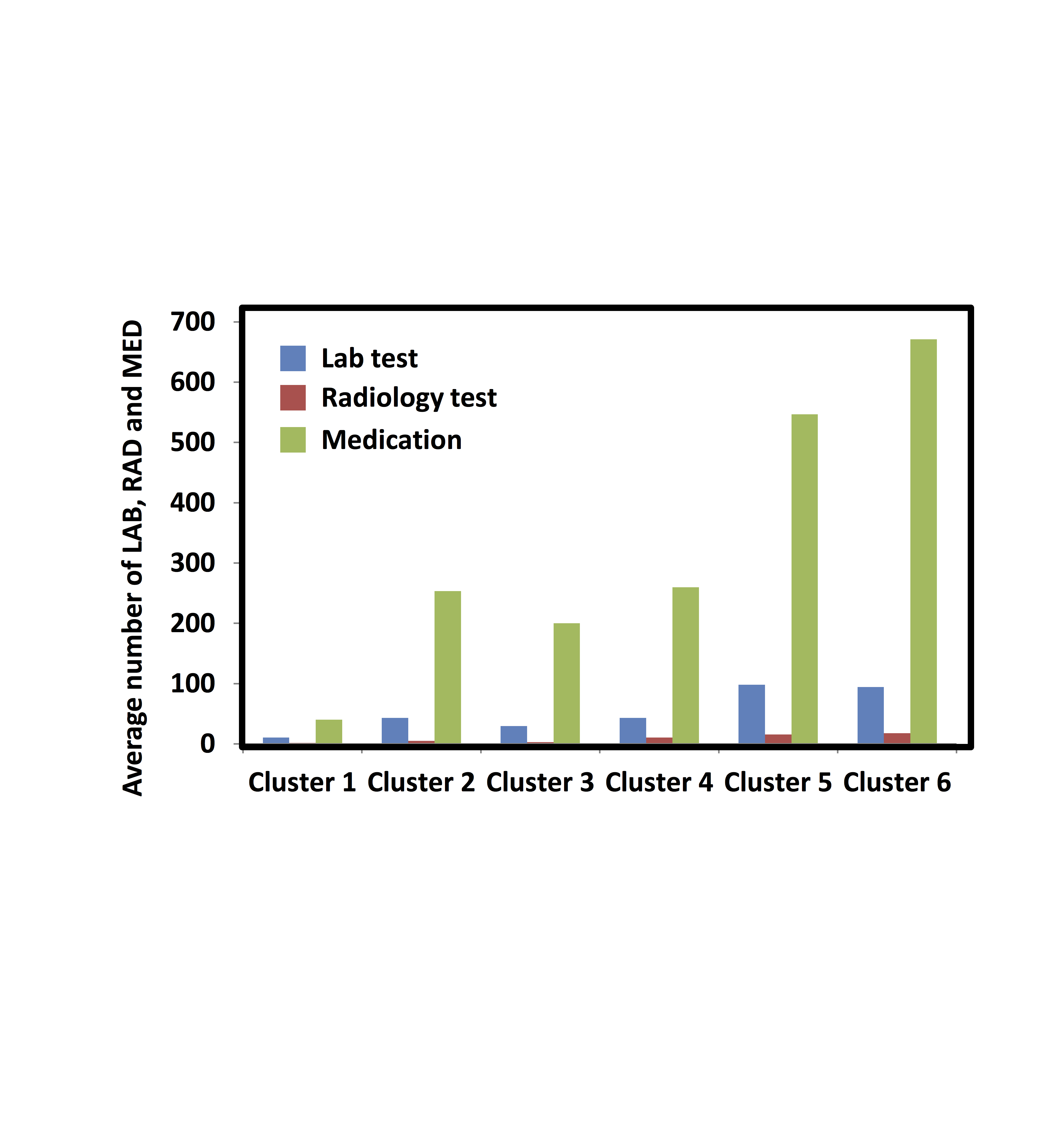
**

**(A)**

**
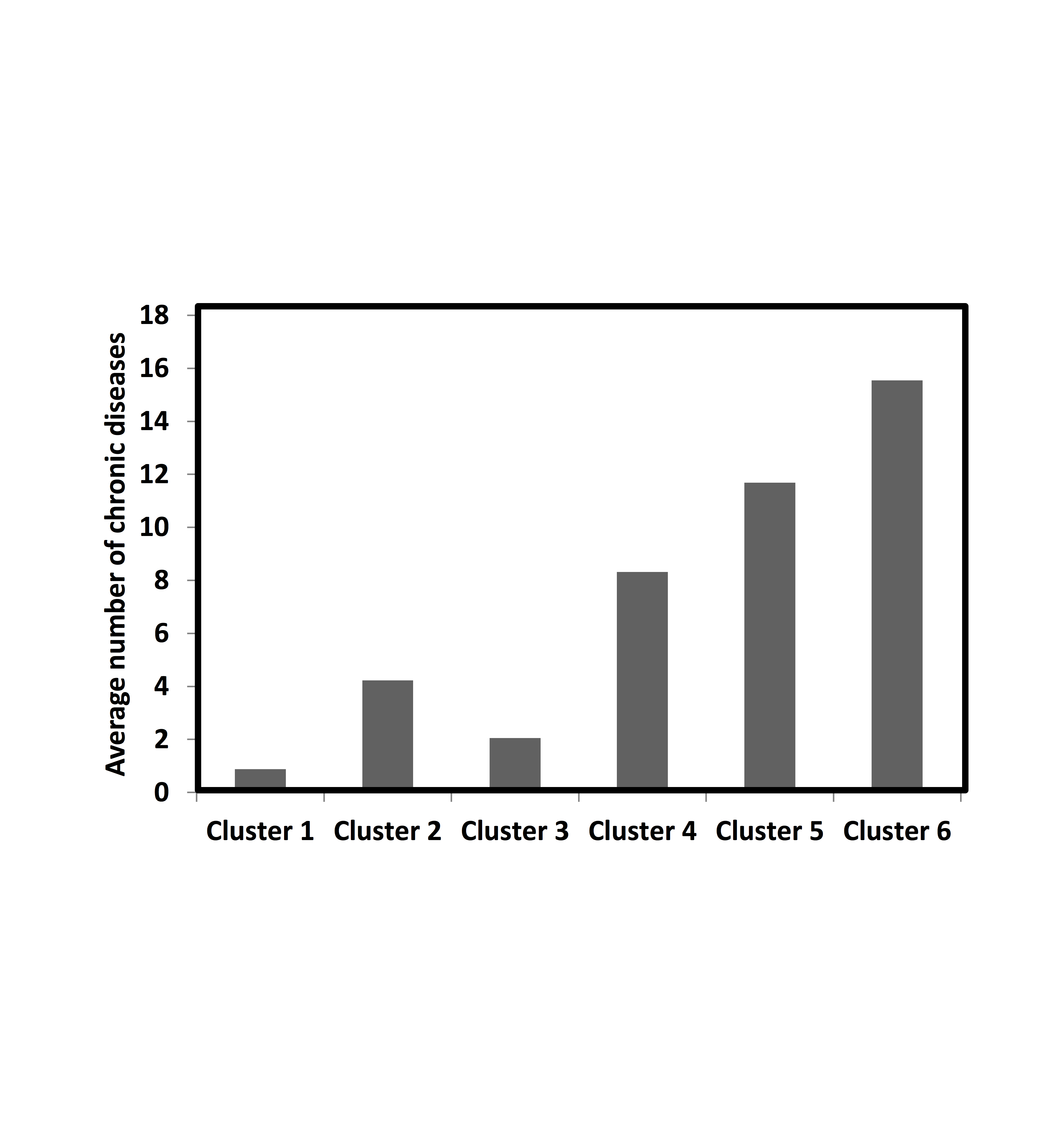
**

**(B)**

**
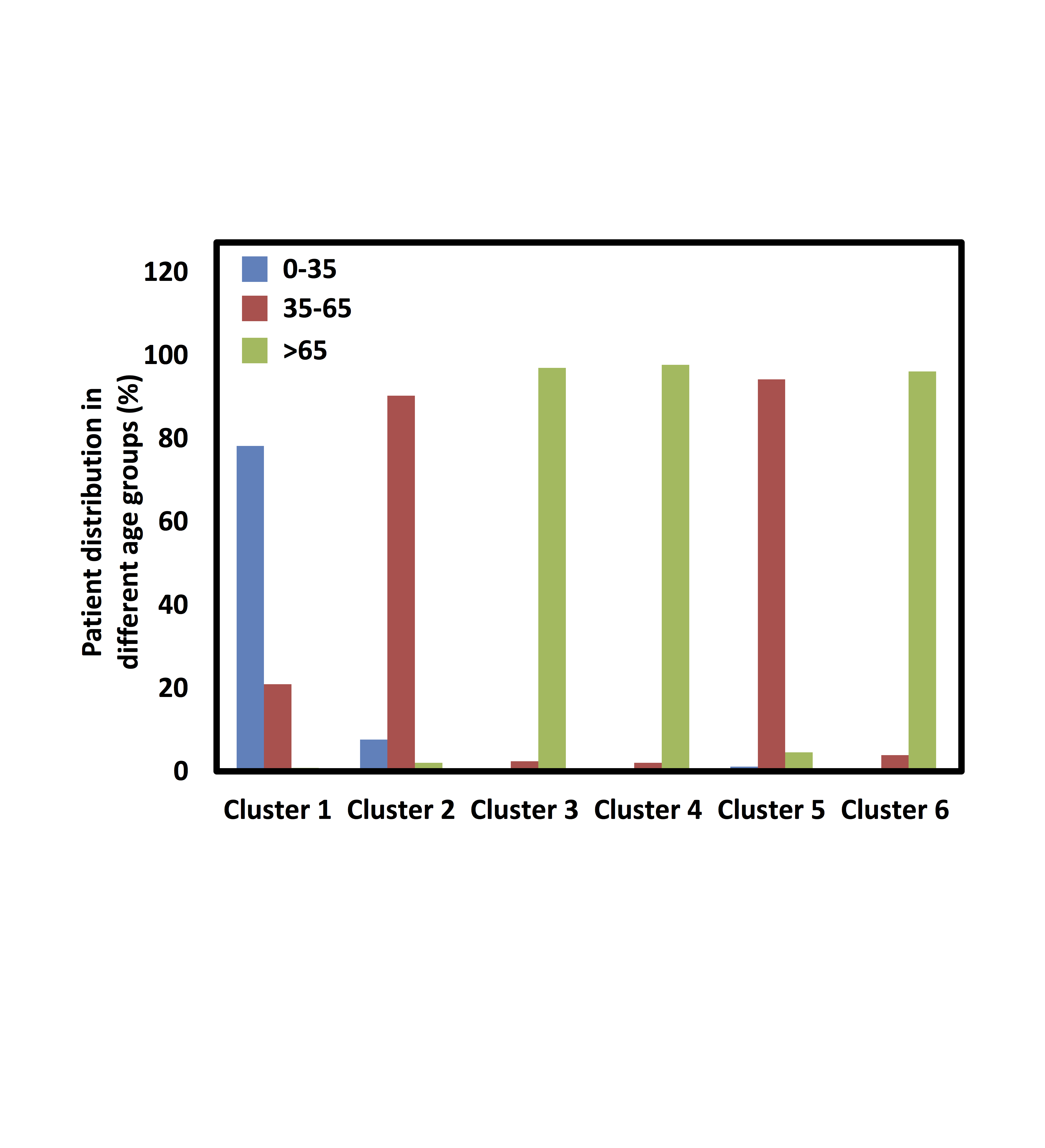
**

**(C)**

**
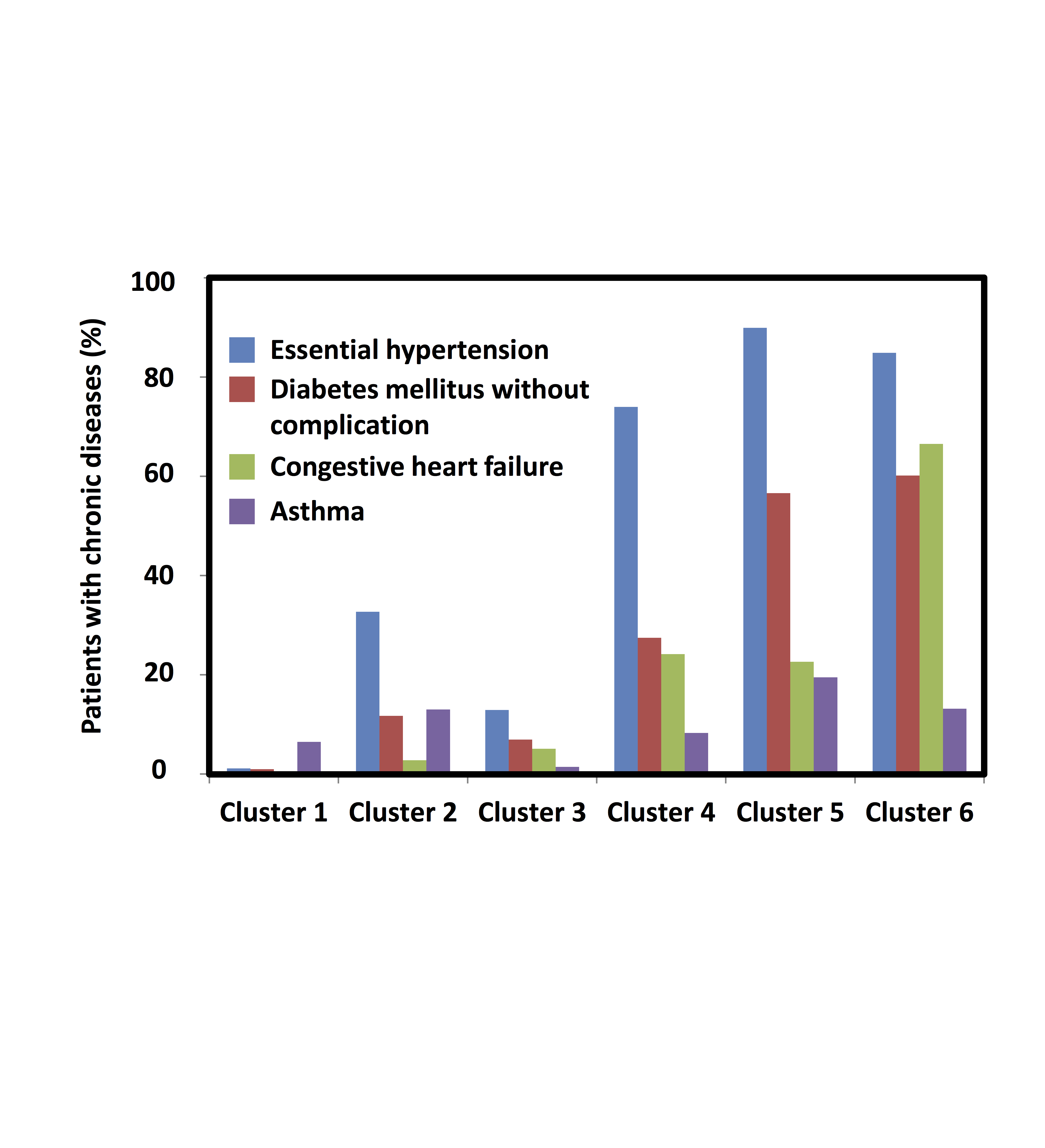
**

**(D)**

**S6 Fig.** The prospective unsupervised learning of high-risk encounters. Summary of clinical patterns in each cluster is shown in (A)-(D). The y-axes stand for (A) the average number of lab tests, radiographic studies, and medications; (B) the average number of chronic diseases; (C) the percentage of three age groups, and (D) the percentage of chronic diseases.
