# Supplementary material for: Development, Validation and Deployment of a Real Time 30 Day Hospital Readmission Risk Assessment Tool in the Maine Healthcare Information Exchange
Source: PLoS One. 2015 Oct 8;10(10):e0140271. doi: 10.1371/journal.pone.0140271 (PMC4598005; doi:10.1371/journal.pone.0140271)
Supplement: S7 Fig — (DOCX) [file pone.0140271.s007.docx]

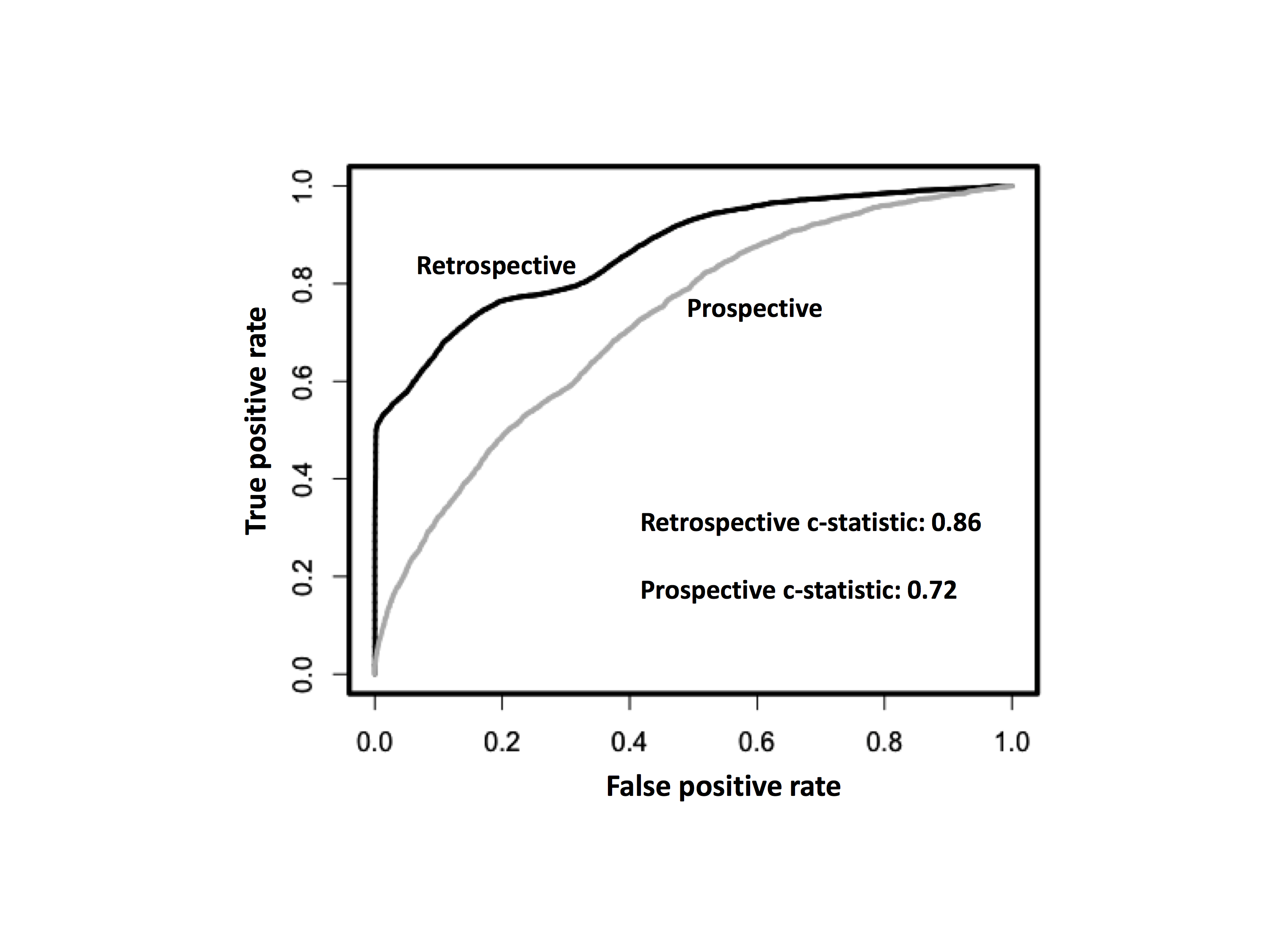


**S7 Fig**. **Binary classification performance of risk scores with retrospective cohort and prospective cohort, respectively.**
