# Supplementary material for: Development, Validation and Deployment of a Real Time 30 Day Hospital Readmission Risk Assessment Tool in the Maine Healthcare Information Exchange
Source: PLoS One. 2015 Oct 8;10(10):e0140271. doi: 10.1371/journal.pone.0140271 (PMC4598005; doi:10.1371/journal.pone.0140271)
Supplement: S1 File — (DOCX) [file pone.0140271.s008.docx]

**S1 File. Data warehouse**

Data used for this study were directly collected by the Maine’s HIE ORION/IBM Initial/CDC database which managed real-time data transition (HL-7 message transition). The Maine HIE went live in 2009 and now contains records for close to all of Maine residents and is connected to the majority of health care facilities in Maine. The HIE includes records for over 1 million individuals including in-state and out-of-state residents. Over 90% of Maine residents have a record in the database. Data associated with each record were encounter based, and most of the data were counts from the whole admission period. HIN is an independent, nonprofit organization operating the HIE in Maine. It maintains an opt-out consent process for general medical information and an opt-in patient consent for certain behavioral health and HIV related information as required by Maine State law. The HIE has just over a 1% patient opt-out rate.

Incorporated data elements from EMR encounters include patient demographic information, laboratory tests and results, radiographic procedures, medication prescriptions, primary and secondary diagnoses and procedures which are coded according to the International Classification of Diseases, 9th Revision, Clinical Modification (ICD-9-CM). Primary and secondary diagnoses were further clustered into 190 chronic diagnoses according to the Clinical Classifications Software (CCS) Developed at the Agency for Healthcare Research and Quality (AHRQ). Comorbidities were described by those CCS-coded diagnoses. Census data from the U.S. Department of Commerce Census Bureau were integrated into our data warehouse, to provide approximation on patients’ socioeconomic status information in terms of the average household mean and median family income and average degree of educational attainment, based on residence zip codes.
