# Supplementary material for: Development, Validation and Deployment of a Real Time 30 Day Hospital Readmission Risk Assessment Tool in the Maine Healthcare Information Exchange
Source: PLoS One. 2015 Oct 8;10(10):e0140271. doi: 10.1371/journal.pone.0140271 (PMC4598005; doi:10.1371/journal.pone.0140271)
Supplement: S2 File — (DOCX) [file pone.0140271.s009.docx]

**S2 File. Missing data handling**

Inevitably some data were missing for some records in our data warehouse. It was a data integrity problem that existed before we obtained data from HIE. There were around 3.4% of encounters with missing geographic information, 0.68% with missing gender, and 0.20% with missing admission or discharge date during 2012 and 2013 in our database. Encounter records with missing demographics were removed from our study cohort. There were possibly some longitudinal data of clinical histories missing as well. However, it was hard to tell from the database whether such information was actually missing or not. For example, having an inpatient admission count of 0 in prior one year may indicate that the patient didn’t visit a hospital during last year, or the admission information for this patient was not transferred to the HIE (though it would happen at a very low probability). To deal with this potential problem, we used natural language processing techniques to extract diagnosis, lab test results, and medication information from clinical notes to enrich our database.
