# Supplementary material for: Development, Validation and Deployment of a Real Time 30 Day Hospital Readmission Risk Assessment Tool in the Maine Healthcare Information Exchange
Source: PLoS One. 2015 Oct 8;10(10):e0140271. doi: 10.1371/journal.pone.0140271 (PMC4598005; doi:10.1371/journal.pone.0140271)
Supplement: S1 Table — (DOCX) [file pone.0140271.s010.docx]

| **S1 Table. Patient characteristics** | | | | |
| --- | --- | --- | --- | --- |
|  | **Retrospective** | | **Prospective** | |
|  | **(Jan.1, 2012 – Dec.31, 2012)** | | **(Jan.1, 2013 – Dec.31, 2013)** | |
|  | **Control** | **Case** | **Control** | **Case** |
|  | **N = 65,790** | **N = 8,694** | **N = 103,721** | **N = 15,230** |
| **Gender** | | | | |
| Female | 56.44% | 54.45% | 55.94% | 52.58% |
| Male | 43.56% | 45.55% | 44.06% | 47.42% |
| **Age** | | | | |
| Median(IQR) | 60.69 | 66,87 | 60.28 | 65.29 |
|  | (33.96, 76.14) | (51.44, 79.23) | (32.50, 75.56) | (48.59, 78.05) |
| **Median family income estimate** | | | | |
| Median(IQR) | 55610 | 55313 | 58054 | 56389 |
|  | (48819, 63440) | (48750, 63440) | (49128, 66484) | (48839, 64000) |
| **Percent high school graduate or higher** | | | | |
| Median(IQR) | 89.46 | 89.40 | 90.00 | 89.50 |
|  | (86.40, 91.80) | (86.40, 91.47) | (86.90, 92.50) | (86.50, 92.00) |
| **Percent bachelor's degree or higher** | | | | |
| Median(IQR) | 22.20 | 22.20 | 23.45 | 22.60 |
|  | (16.60, 28.30) | (16.70, 28.30) | (17.70, 29.10) | (17.20, 29.00) |
